# Atherosclerosis index and BMI: new predictors of cognitive function in ischemic survivors

**DOI:** 10.3389/fnut.2025.1703425

**Published:** 2025-11-12

**Authors:** Lingyan Zhao, Chenyang Qin, Hanbo Yu, Luofan Zhang, Dingchen Zhang, Shu Wang, Guiping Li

**Affiliations:** 1 First Teaching Hospital of Tianjin University of Traditional Chinese Medicine, Tianjin, China; 2 National Clinical Research Center for Chinese Medicine Acupuncture and Moxibustion, Tianjin, China; 3 Tianjin Academy of Traditional Chinese Medicine Affiliated Hospital, Tianjin, China

**Keywords:** ischemic stroke, atherogenic index of plasma, insulin resistance, metabolism, cognitive function

## Abstract

**Background:**

The atherogenic index of plasma (AIP) is a reliable surrogate marker for insulin resistance and is strongly associated with both stroke risk and prognosis. However, the associations of AIP and the composite index AIP-BMI with cognitive function among patients with ischemic stroke remain insufficiently studied.

**Methods:**

This cross-sectional study included 2,933 patients with ischemic stroke. Demographic and clinical data were collected from all participants. The AIP was calculated as log [TG (mmol/L)/HDL-C (mmol/L)], and cognitive function was evaluated using the Mini-Mental State Examination (MMSE). Multivariable linear regression models were applied to examine the associations between AIP (and AIP-BMI) and MMSE scores, adjusting for potential confounders. Stratified and sensitivity analyses were further conducted to evaluate the robustness of the findings.

**Results:**

The mean age of participants was 64.8 years (SD 10.2), and 2,009 (68.5%) were male. Each one-unit increase in AIP was associated with a 1.15-point reduction in MMSE score (*p* < 0.001). Similarly, each one-unit increase in AIP-BMI corresponded to a 0.04-point decrease in MMSE score (*p* < 0.001). The inverse associations remained consistent when AIP and AIP-BMI were analyzed by tertiles.

**Conclusion:**

Higher levels of AIP and AIP-BMI are independently associated with poorer cognitive performance in patients with ischemic stroke. These findings suggest that dyslipidemia-related metabolic disturbances may contribute to post-stroke cognitive impairment.

**Clinical trial registration:**

https://www.chictr.org.cn/showproj.html?proj=120858, identifier ChiCTR2100042721.

## Introduction

Stroke remains the leading cause of death and disability worldwide, with ischemic stroke (IS) accounting for approximately 87% of all stroke cases (1). According to the Global Burden of Disease Study (GBD) 2021, the incidence and recurrence rates of IS have been steadily increasing, particularly in developing countries and aging populations (2). Despite remarkable advances in acute stroke management, such as intravenous thrombolysis and endovascular therapy (3–5), a substantial proportion of survivors experience persistent neurological and functional impairments (6, 7). Post-stroke cognitive impairment (PSCI) is one of the most common and disabling sequelae to stroke. Epidemiological studies have reported that the prevalence of PSCI ranges from 24 to 53.4% (8, 9). Moreover, PSCI markedly increases long-term mortality risk, with patients who develop post-stroke dementia exhibiting mortality rates two to five times higher than those without dementia (10, 11). Therefore, early identification and risk stratification of PSCI are of critical importance for improving long-term outcomes for stroke survivors.

The atherogenic index of plasma (AIP), first proposed by Dobiasova et al., represents the logarithmic ratio of triglycerides (TG) to high-density lipoprotein cholesterol (HDL-C) (12). AIP not only reflects abnormalities in lipid metabolism and atherosclerotic risk but is also strongly associated with insulin resistance (IR) (13). Increasing evidence indicates that elevated AIP levels are linked to a higher risk of multiple cardiovascular and metabolic disorders, including stroke, myocardial infarction, hypertension, and type 2 diabetes mellitus (14–18). Furthermore, a derived composite index that combines AIP with body mass index (BMI), the AIP-BMI, has been shown to enhance the predictive accuracy for cardiovascular and cerebrovascular risks (19).

Although the clinical significance of AIP has been well established in various disease contexts, most existing studies have primarily focused on its association with stroke incidence and adverse outcomes (14, 20, 21). In contrast, limited research has examined the relationship between AIP and cognitive function following ischemic stroke. Therefore, the present multicenter cross-sectional study aimed to investigate the associations between AIP, AIP-BMI, and cognitive function in patients with ischemic stroke. Using multivariable linear regression and stratified analyses, we sought to determine whether AIP and AIP-BMI could serve as potential early biomarkers of post-stroke cognitive impairment. This study also aims to provide a theoretical basis for the early identification and long-term management of cognitive impairment in stroke survivors, and to identify potential metabolic intervention targets for cognitive rehabilitation.

## Methods

### Study design and population

This cross-sectional study enrolled 3,860 patients diagnosed with ischemic stroke between January 2020 and December 2022 from 27 participating hospitals, including the First Affiliated Hospital of Tianjin University of Traditional Chinese Medicine and Tianjin Huanhu Hospital. A complete list of collaborating centers is provided in Supplementary materials. The study protocol was approved by the Ethics Committee of the First Affiliated Hospital of Tianjin University of Traditional Chinese Medicine (Approval No.: TYLL2021[K]015). Written informed consent was obtained from all participants before enrollment, and the study was conducted in accordance with the principles of the Declaration of Helsinki.

The diagnosis of ischemic stroke was based on clinical manifestations and neuroimaging findings, following the diagnostic criteria established by the Neurology Branch of the Chinese Medical Association for ischemic cerebral infarction (22). Stroke subtypes were classified according to the Oxford Community Stroke Project (OCSP) classification system (23).

The inclusion criteria were as follows: (1) age ≥ 35 years, (2) fulfillment of the clinical and imaging diagnostic criteria for ischemic stroke, and (3) time from stroke onset to cognitive assessment ≤ 3 months, with a stable condition sufficient to complete the cognitive evaluation.

**Figure 1:**
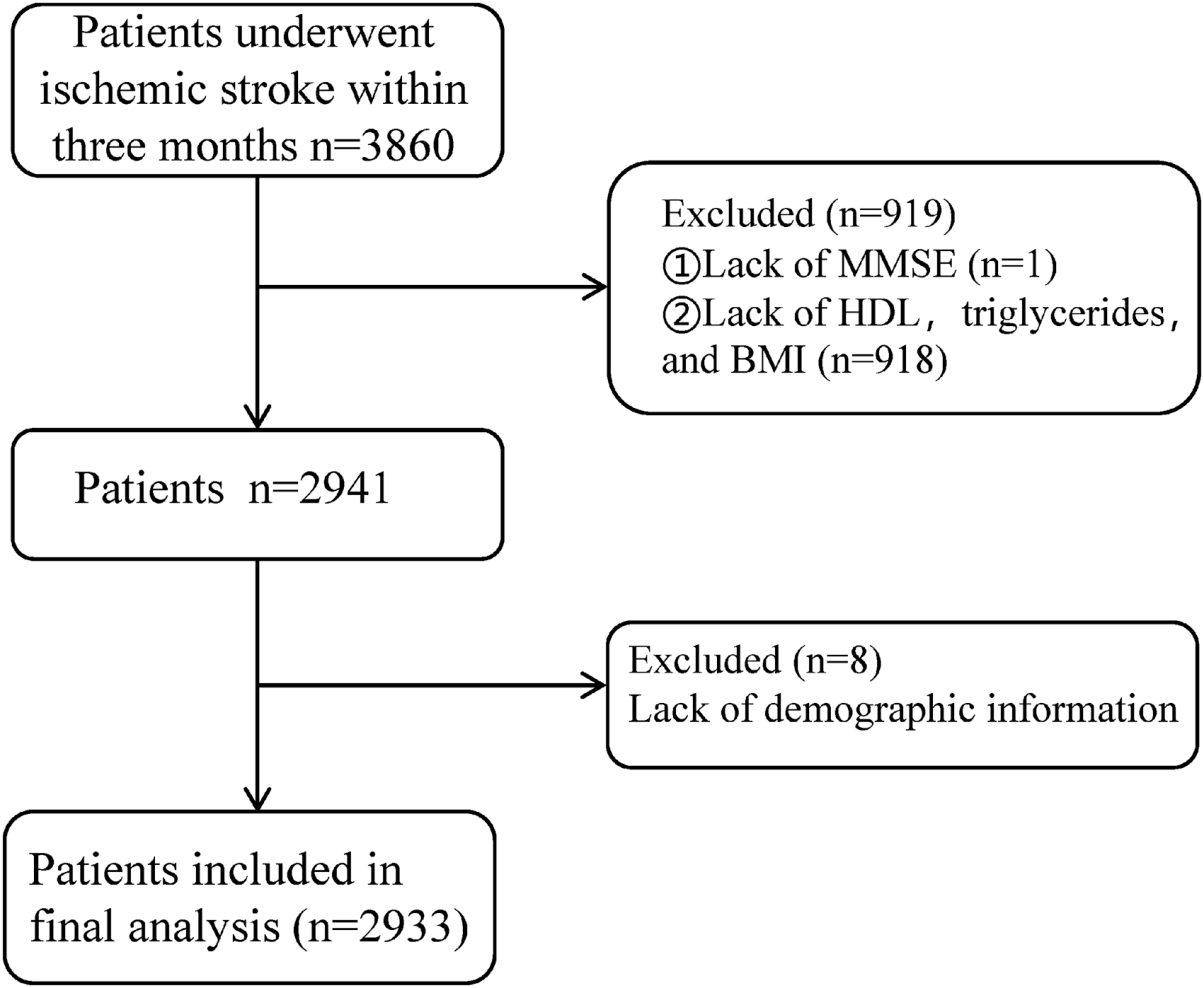
Flowchart of the selection of IS patients.

The exclusion criteria were: (1) missing primary outcome data (Mini-Mental State Examination, MMSE scores, *n* = 1); (2) extreme outliers in lipid levels or BMI (greater than three times the interquartile range) or missing data (*n* = 918); (3) missing demographic information (*n* = 8); (4) presence of severe psychiatric disorders, impaired consciousness, or aphasia precluding MMSE assessment; and (5) serious systemic diseases, including end-stage liver or kidney disease, or malignancies. After applying these criteria, 2,933 participants were ultimately included in the final analysis (Figure 1).

### Data collection

Demographic and clinical information, including age, sex, ethnicity, marital status, years of education, occupation, smoking status, BMI, and alcohol consumption, was collected from all participants. Laboratory parameters, including TG, HDL-C, low-density lipoprotein cholesterol (LDL-C), total cholesterol (TC), and fasting blood glucose (FBG), were assessed within 24 h of hospital admission. Additionally, a detailed family medical history was documented, including whether either parent had a history of hypertension, coronary heart disease, or diabetes mellitus. Comorbidities included hypertension, diabetes mellitus, coronary heart disease, atrial fibrillation, and previous stroke, with detailed diagnostic criteria provided in Supplementary materials.

### Cognitive function assessment

Cognitive function after ischemic stroke was assessed using the MMSE, a version adapted for Chinese populations by Katzman et al. (24). Evaluations were conducted once patients’ clinical conditions were stable, and all assessments were administered by trained neuropsychological evaluators who completed standardized certification prior to the study.

### Definitions of AIP and AIP-BMI

The AIP, designated as the primary research variable, is calculated using the formula: AIP = log [TG (mmol/L)/HDL-C (mmol/L)]. AIP-BMI is derived using the equation AIP × BMI. Participants were categorized into three groups based on tertiles: Group T1 (AIP < 0.011), Group T2 (0.011 ≤ AIP < 0.499), and Group T3 (AIP ≥ 0.499).

### Statistical analysis

Ischemic stroke patients were divided into tertiles based on AIP values, designated as Group T1, Group T2, and Group T3. The distribution characteristics of continuous variables were first evaluated using the Shapiro–Wilk test. For variables that approximated a normal distribution, group differences were compared using one-way analysis of variance (ANOVA), and results were expressed as mean ± standard deviation (SD). For non-normally distributed variables, comparisons were conducted using the Kruskal–Wallis rank-sum test, and data were presented as median (interquartile range, IQR). Categorical variables were summarized as frequencies and percentages (%) and compared using the chi-squared test.

The AIP and AIP-BMI indices were analyzed both as continuous and categorical variables (based on tertiles) to evaluate their associations with Mini-Mental State Examination (MMSE) scores among ischemic stroke patients. These associations were assessed by calculating effect estimates (*β*) and 95% confidence intervals (CIs). Potential confounding variables were selected based on prior literature and clinical relevance (25, 26). Analyses were performed using three hierarchical models: Model 1: unadjusted; Model 2: adjusted for age, education level, smoking status, LDL-C, and duration of illness; and Model 3: further adjusted for family history of hypertension, presence of hypertension, atrial fibrillation, and OCSP classification (total anterior circulation infarct and lacunar infarct types).

Residual analyses were performed to assess model fit and normality, while variance inflation factor (VIF) testing was used to evaluate multicollinearity. To examine potential non-linear dose–response relationships between AIP and MMSE scores, restricted cubic spline (RCS) regression models were applied, adjusting for all covariates included in Model 3.

To evaluate the robustness of the associations, subgroup analyses were conducted according to age (≤65 vs. >65 years), sex (male vs. female), hypertension (no vs. yes), and diabetes mellitus (no vs. yes). The likelihood ratio test was used to assess interactions between subgroups. Additionally, sensitivity analyses were carried out to evaluate the impact of extreme outliers on the robustness of the findings. All statistical analyses were performed using R software (version 4.5.0). All reported *p*-values were two-tailed, and a *p*-value of < 0.05 was considered statistically significant.

## Results

This study initially included 3,860 patients diagnosed with ischemic stroke. After excluding 927 cases due to extreme outlier measurements and missing data, a total of 2,933 patients were ultimately included. Continuous missing data were predicted and imputed using a linear regression model. The average age of the included patients was 64.8 years, with 2,009 men (68.5%) and 924 women (31.5%). Group T1 (AIP < 0.011) comprised 978 patients, Group T2 (0.011 ≤ AIP < 0.499) included 977 patients, and Group T3 (AIP ≥ 0.499) consisted of 978 patients (see Table 1).

**Table 1:** Characteristics of ischemic stroke patients by categories of AIP.

Variables	T	T1	T2	T3	*p*
	(*N* = 2,933)	(*N* = 978)	(*N* = 977)	(*N* = 978)	
Age (years, M + SD)	64.8 (10.2)	67.3 (10.1)	64.3 (9.87)	62.7 (10.2)	0.008
Sex					0.005
Male	2009 (68.5%)	633 (64.7%)	690 (70.6%)	686 (70.1%)	
Female	924 (31.5%)	345 (35.3%)	287 (29.4%)	292 (29.9%)	
Region area					0.864
North	2,248 (76.6%)	745 (76.2%)	748 (76.6%)	755 (77.2%)	
South	685 (23.4%)	233 (23.8%)	229 (23.4%)	223 (22.8%)	
Ethnicity					0.656
Han ethnic	2,846 (97.0%)	947 (96.8%)	952 (97.4%)	947 (96.8%)	
Others	87 (3.0%)	31 (3.2%)	25 (2.6%)	31 (3.2%)	
Education					0.001
Illiterate	168 (5.7%)	72 (7.4%)	48 (4.9%)	48 (4.9%)	
Primary school	564 (19.2%)	222 (22.7%)	178 (18.2%)	164 (16.8%)	
Middle school or above	1,187 (40.5%)	370 (37.8%)	402 (41.1%)	415 (42.4%)	
High school or above	1,014 (34.6%)	314 (32.1%)	349 (35.7%)	351 (35.9%)	
Occupation					<0.001
Retirement	1,559 (53.2%)	529 (54.1%)	528 (54.0%)	502 (51.3%)	
Mental work	375 (12.8%)	94 (9.6%)	121 (12.4%)	160 (16.4%)	
Physical work	587 (20.0%)	233 (23.8%)	186 (19.0%)	168 (17.2%)	
Others	412 (14.0%)	122 (12.5%)	142 (14.5%)	148 (15.1%)	
Marital status					0.036
Married	2,643 (90.1%)	865 (88.4%)	879 (90.0%)	899 (91.9%)	
Others	290 (9.9%)	113 (11.6%)	98 (10.0%)	79 (8.1%)	
Smoke status					0.003
Never smoked	1,608 (54.8%)	584 (59.7%)	514 (52.6%)	510 (52.1%)	
Former smoker	463 (15.8%)	139 (14.2%)	170 (17.4%)	154 (15.7%)	
Current smoker	862 (29.4%)	255 (26.1%)	293 (30.0%)	314 (32.1%)	
Alcohol status					0.049
Never drank	1,676 (57.1%)	590 (60.3%)	546 (55.9%)	540 (55.2%)	
Former drinker	361 (12.3%)	111 (11.3%)	112 (11.5%)	138 (14.1%)	
Current drinker	896 (30.5%)	277 (28.3%)	319 (32.7%)	300 (30.7%)	
BMI	23.8 (3.09)	24.8 (3.11)	25.2 (3.21)	24.6 (3.19)	<0.001
Weight	69.1 (11.0)	66.3 (10.9)	69.8 (10.5)	71.2 (11.2)	<0.001
Height	167 (7.63)	167 (7.74)	168 (7.53)	168 (7.56)	0.001
Course of disease	20.0 (20.4)	19.0 (20.5)	19.8 (20.2)	21.1 (20.5)	0.001
Recurrent_stroke					0.517
No	2034 (69.3%)	691 (70.7%)	675 (69.1%)	668 (68.3%)	
Yes	899 (30.7%)	287 (29.3%)	302 (30.9%)	310 (31.7%)	
Location of cerebral infarction					
TACI					0.200
No	2,815 (96.0%)	945 (96.6%)	929 (95.1%)	941 (96.2%)	
Yes	118 (4.0%)	33 (3.4%)	48 (4.9%)	37 (3.8%)	
PACI					0.657
No	1,422 (48.5%)	471 (48.2%)	485 (49.6%)	466 (47.6%)	
Yes	1,511 (51.5%)	507 (51.8%)	492 (50.4%)	512 (52.4%)	
POCI					0.965
No	2,201 (75.0%)	735 (75.2%)	735 (75.2%)	731 (74.7%)	
Yes	732 (25.0%)	243 (24.8%)	242 (24.8%)	247 (25.3%)	
LACI					0.678
No	2,106 (71.8%)	695 (71.1%)	699 (71.5%)	712 (72.8%)	
Yes	827 (28.2%)	283 (28.9%)	278 (28.5%)	266 (27.2%)	
Family medical history					
HTN					0.317
No	2,436 (83.1%)	824 (84.3%)	813 (83.2%)	799 (81.7%)	
Yes	497 (16.9%)	154 (15.7%)	164 (16.8%)	179 (18.3%)	
DM					0.006
No	2,718 (92.7%)	927 (94.8%)	900 (92.1%)	891 (91.1%)	
Yes	215 (7.3%)	51 (5.2%)	77 (7.9%)	87 (8.9%)	
CHD					0.100
No	2,838 (96.8%)	956 (97.8%)	940 (96.2%)	942 (96.3%)	
Yes	95 (3.2%)	22 (2.2%)	37 (3.8%)	36 (3.7%)	
Stroke					0.959
No	2,605 (88.8%)	867 (88.7%)	870 (89.0%)	868 (88.8%)	
Yes	328 (11.2%)	111 (11.3%)	107 (11.0%)	110 (11.2%)	
Co-morbidities					
HTN					0.002
No	899 (30.7%)	329 (33.6%)	311 (31.8%)	259 (26.5%)	
Yes	2034 (69.3%)	649 (66.4%)	666 (68.2%)	719 (73.5%)	
DM					<0.001
No	1825 (62.2%)	689 (70.4%)	612 (62.6%)	524 (53.6%)	
Yes	1,108 (37.8%)	289 (29.6%)	365 (37.4%)	454 (46.4%)	
CHD					0.872
No	2,387 (81.4%)	797 (81.5%)	799 (81.8%)	791 (80.9%)	
Yes	546 (18.6%)	181 (18.5%)	178 (18.2%)	187 (19.1%)	
AF					0.829
No	2,816 (96.0%)	937 (95.8%)	937 (95.9%)	942 (96.3%)	
Yes	117 (4.0%)	41 (4.2%)	40 (4.1%)	36 (3.7%)	
Surgical history					0.796
No	2,174 (74.1%)	718 (73.4%)	725 (74.2%)	731 (74.7%)	
Yes	759 (25.9%)	260 (26.6%)	252 (25.8%)	247 (25.3%)	
Blood biochemistry					
Fasting blood glucose	7.17 (23.1)	7.54 (36.4)	7.14 (16.2)	6.84 (2.47)	<0.001
Triglycerides	1.42 (0.64)	0.904 (0.28)	1.32 (0.29)	2.04 (0.64)	<0.001
Low-density lipoprotein	2.33 (0.89)	2.21 (0.87)	2.39 (0.89)	2.40 (0.91)	<0.001
High-density lipoprotein	1.23 (4.25)	1.81 (7.32)	1.02 (0.21)	0.852 (0.20)	<0.001
Cholesterol	4.27 (10.3)	3.96 (3.36)	4.36 (10.8)	4.50 (13.9)	0.104
Primary outcome					
MMSE	23.1 (7.54)	23.7 (7.07)	23.0 (7.57)	22.7 (7.93)	0.008

Data are represented as mean ± SD or frequency (percent). Statistical comparisons were conducted using the Kruskal–Wallis test for continuous variables and the chi-squared test for categorical variables.

BMI, body mass index; TACI, total anterior circulation infarction; PACI, partial anterior circulation infarction; POCI, posterior circulation infarction; HTN, hypertension; DM, diabetes mellitus; CHD, coronary heart disease; AF, atrial fibrillation.

Patients in the high AIP group were younger, had a higher proportion of males, and exhibited elevated levels of height, weight, triglycerides, and low-density lipoprotein cholesterol (LDL-C) (all *p* < 0.05). Conversely, they displayed lower levels of body mass index (BMI), fasting blood glucose, and high-density lipoprotein cholesterol (HDL-C). Moreover, high AIP levels were significantly associated with increased incidence rates of hypertension and diabetes, as well as a higher prevalence of a family history of diabetes (all *p* < 0.05). The high AIP group also demonstrated a greater proportion of individuals with higher educational levels, tobacco use, and sedentary occupations (all *p* < 0.05). Furthermore, this group exhibited a longer duration of illness and lower MMSE scores (all *p* < 0.05).

**Figure 2:**
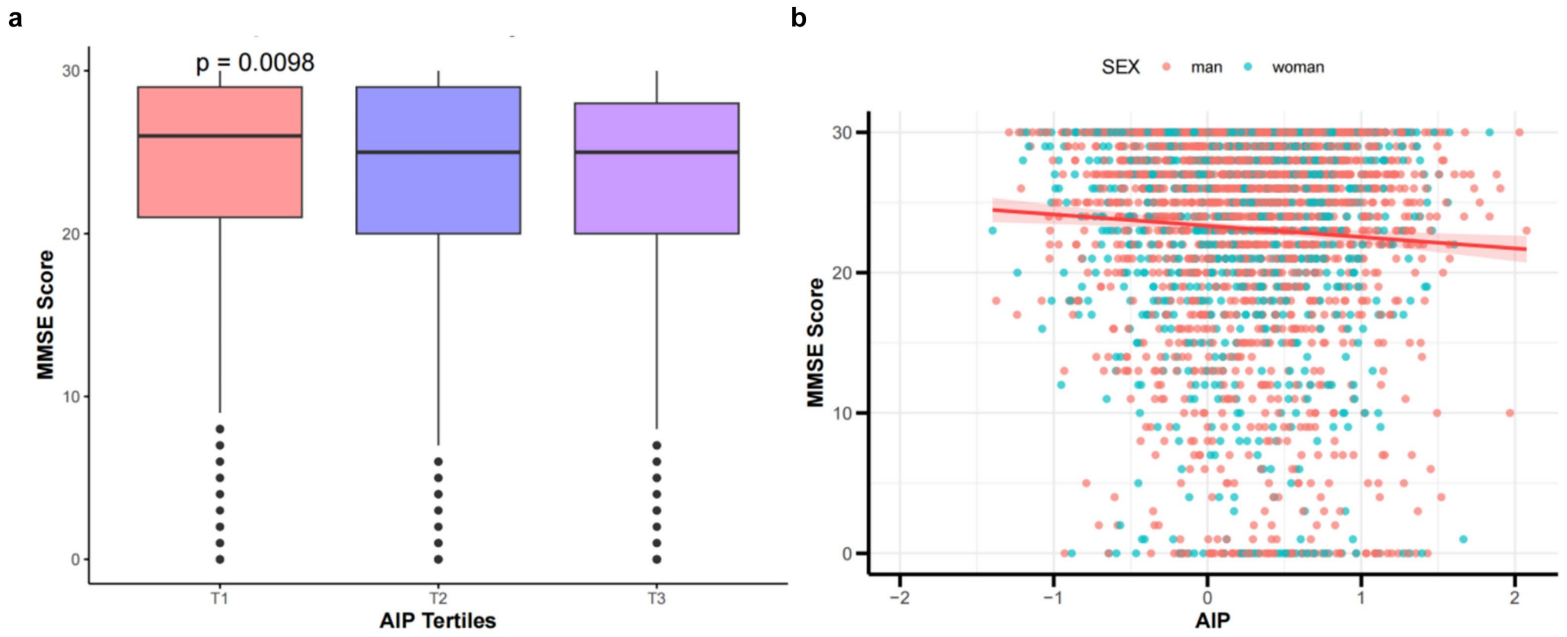
**(a)** Comparison of MMSE scores by tertile groups of the AIP. **(b)** Scatter plot shows the relationship between MMSE scores and AIP in patients with IS. The X-axis denotes the AIP, where each division corresponds to 1 unit. The Y-axis indicates MMSE scores, with each division representing 10 points.

This study generated histograms and scatter plots for patients with ischemic stroke, indicating that as AIP increases, there is a significant downward trend in MMSE scores, as illustrated in Figures 2a,b. Additionally, an increase in AIP-BMI is significantly associated with lower MMSE scores (see Supplementary Figure S1).

Linear regression analyses were conducted to examine the relationships between AIP, AIP-BMI, and MMSE scores in patients with ischemic stroke. Both AIP and AIP-BMI were analyzed as continuous and categorical variables, using Group T1 as the reference. The associations between AIP, AIP-BMI, and MMSE scores were assessed through simple and multiple linear regression analyses. In the unadjusted Model 1, both AIP and AIP-BMI were associated with reductions in MMSE scores (*p* ≤ 0.05). After adjusting for confounding factors, AIP and AIP-BMI remained significantly correlated with MMSE scores. Specifically, for each one-unit increase in AIP, MMSE scores decreased by 1.15 points (*p* < 0.001); participants in the highest tertile had MMSE scores that were 1.55 points lower than those in the lowest tertile (*p* < 0.001) (see Table 2). Similarly, for each one-unit increase in AIP-BMI, MMSE scores decreased by 0.04 points (*p* < 0.001); compared to participants in the lowest tertile, those in the highest tertile had MMSE scores that were 1.41 points lower (*p* < 0.001) (see Table 2).

**Table 2:** Linear regression analysis of the AIP and AIP-BMI in relation to cognitive function in patients with ischemic stroke.

Variables	Model 1		Model 2		Model 3	
	β(95%CI)	*p*	β(95%CI)	*p*	β(95%CI)	*p*
AIP	−0.80(−1.27 to −0.34)	<0.001	−1.28(−1.74 to −0.83)	<0.001	−1.15(−1.60 to −0.70)	<0.001
T1	ref		Ref		ref	
T2	−0.73(−1.40 to −0.06)	0.03	−1.27(−1.92 to −0.62)	<0.001	−1.16(−1.79 to −0.52)	<0.001
T3	−1.0(−1.67–0.34)	0.003	−1.72(−2.37 to −1.07)	<0.001	−1.55(−2.20 to −0.91)	<0.001
P for trend		0.003		<0.001		<0.001
AIP-BMI	−0.03(−0.05 to −0.01)	0.002	−0.05(−0.07 to −0.03)	<0.001	−0.04(−0.06 to −0.03)	<0.001
T1	ref		ref		ref	
T2	−0.97(−1.64 to −0.3)	0.005	−1.41(−2.06 to −0.77)	<0.001	−1.30(−1.93 to −0.66)	<0.001
T3	−0.78(−1.45 to −0.12)	0.02	−1.58(−2.23 to −0.92)	<0.001	−1.41(−2.06 to −0.77)	<0.001
P for trend		0.02		<0.001		<0.001

Model 1: unadjusted; Model 2: adjusted for age, education, smoking status, LDL-C, and duration of illness; Model 3: further adjusted for family history of HTN, the presence of HTN, AF, and OCSP classification (TACI and LACI).

To further assess the robustness of the models, the VIF for all independent variables was found to be less than 5. The residuals displayed a reasonable distribution, with no significant systematic bias identified. Furthermore, the F-statistics for all models were significant, with *p*-values below 0.05, confirming the overall validity of the models. Therefore, both the goodness-of-fit and the validity of the models were substantiated, reinforcing the significant relationship between AIP, AIP-BMI, and MMSE scores. Furthermore, the results of this study indicate that the AIC and BIC values in the model using AIP were slightly higher than those in the model incorporating AIP-BMI, despite controlling for the same confounding factors. This suggests that the latter model offers a more favorable balance between model fit and complexity.

**Figure 3:**
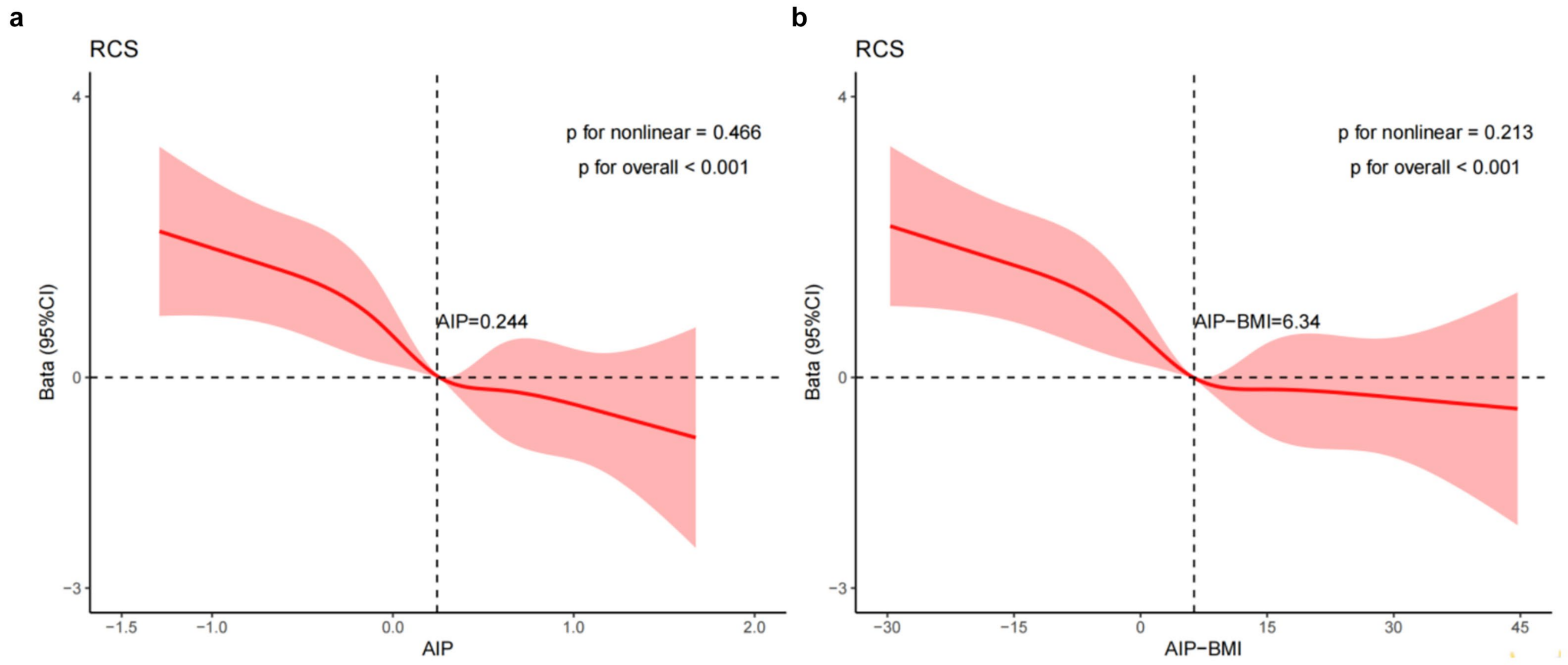
RCS illustrates the relationships between the AIP **(a)** and AIP-BMI **(b)** with MMSE scores, fitted using a linear regression model. This model was adjusted for age, education, smoking status, LDL-C, duration of illness, family history of HTN, the presence of HTN, AF, and OCSP classification (TACI and LACI). The solid line represents the estimated values, while the shaded area indicates the 95% confidence interval. In **(a)** presents the X-axis, which denotes the AIP. In **(b)**, the X-axis indicates the AIP-BMI, while the Y-axis represents the *β* coefficient.

To evaluate the associative relationships among AIP, AIP-BMI, and MMSE scores in patients with ischemic stroke after adjusting for covariates in Model 3, we constructed RCS curves. As shown in Figure 3, a linear relationship exists between AIP levels and MMSE scores in these patients (*p* for non-linearity = 0.466), with an inflection point at AIP equal to 0.244. For values exceeding 0.244, each one-unit increase in AIP is associated with a decrease in MMSE scores. Similarly, there is a linear correlation between AIP-BMI and MMSE scores (*p* for non-linearity = 0.213), with an inflection point at AIP-BMI equal to 6.34. For values above 6.34, each one-unit increase in AIP-BMI corresponds to a decrease in MMSE scores.

### Subgroup analysis

**Figure 4:**
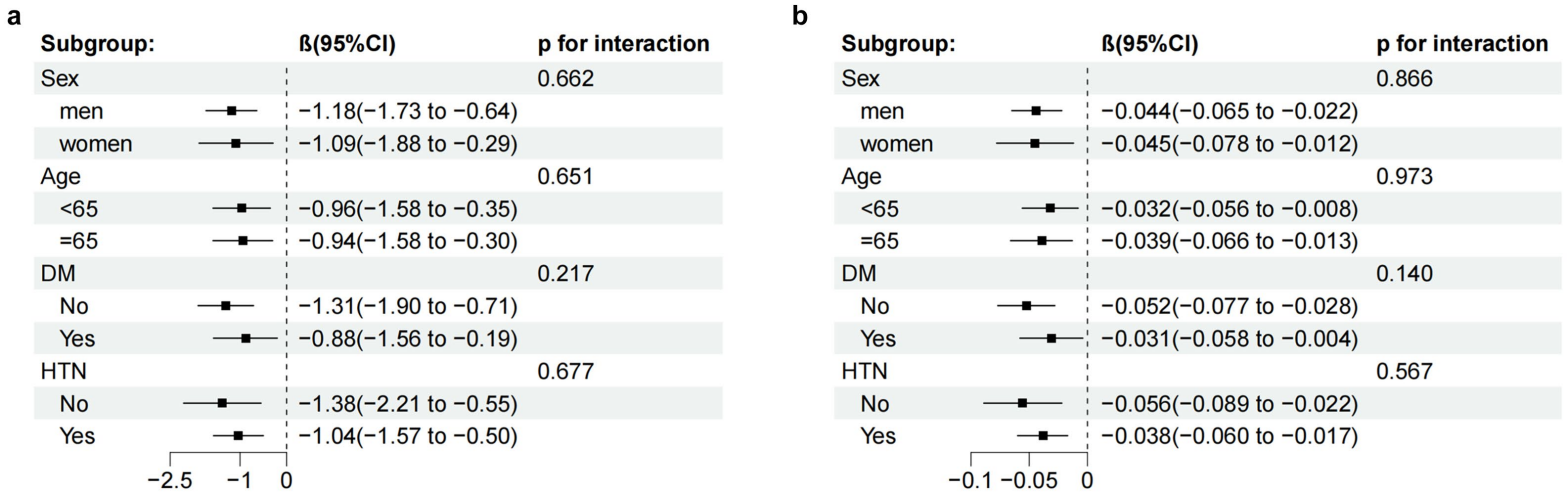
Subgroup analysis of the relationship between the AIP **(a)** AIP-BMI **(b)** and MMSE scores, adjusted according to age, education, smoking status, LDL-C, duration of illness, family history of HTN, the presence of HTN, AF, and OCSP classification (TACI and LACI). CI, confidence interval.

In the subgroup analyses, the relationship between AIP and MMSE scores in patients with ischemic stroke was examined. The computed interaction *p*-values (all *p* > 0.05) indicated no significant interactions. However, within the different subgroups, the AIP index demonstrated statistical significance in predicting reductions in MMSE scores (as shown in Figure 4), highlighting the stability and consistency of the association between AIP and MMSE scores.

Additionally, subgroup analyses of the relationship between AIP-BMI and MMSE scores in patients with ischemic stroke were conducted. Similarly, the computed interaction *p*-values (all *p* > 0.05) did not suggest significant interactions. In all groups, the AIP-BMI index also showed statistical significance in predicting reductions in MMSE scores (as illustrated in Figure 4), further confirming the stability and consistency of the association between AIP-BMI and MMSE scores.

### Sensitivity analysis

To evaluate the robustness of the model results, sensitivity analyses were conducted, specifically examining extreme outliers for TG, HDL-C, and BMI values. In this analysis, patients with extreme outliers for triglycerides, HDL, and BMI (*n* = 78) were not excluded, and their presence did not substantially affect the overall analytical results. Detailed data are provided in Supplementary Table S1, illustrating that the main conclusions remained unchanged, further validating the stability of the model.

## Discussion

This study provides the first evidence of a negative correlation between AIP, AIP-BMI, and cognitive function in patients with ischemic stroke, indicating that as AIP and AIP-BMI increase, MMSE scores decline. In the multiple linear regression analysis, after adjusting for confounding factors such as age, education, smoking status, LDL-C, duration of illness, family history of hypertension, presence of hypertension, atrial fibrillation, and OCSP classification (total anterior circulation infarct type and lacunar infarct type), the correlation remained significant. This suggests that elevated levels of AIP and AIP-BMI may be independently associated with cognitive decline in these patients.

The reliability of these findings was confirmed with trend analysis, stratified analysis, and sensitivity analysis, which indicated no significant interactions among all subgroups, further validating the robustness of the study’s conclusions. Additionally, spline curve fitting revealed a linear relationship between AIP, AIP-BMI, and MMSE decline in patients with ischemic stroke (*p* for non-linearity > 0.05), with inflection points at AIP = 0.244 and AIP-BMI = 6.34.

The AIP, a clinically accessible biomarker of lipid metabolism, is calculated as the logarithmic ratio of TG to HDL-C. It effectively reflects lipid distribution abnormalities and insulin resistance. Numerous studies have confirmed its significant value in assessing the risk of metabolic and cardiovascular diseases. However, given the limitations of AIP as a single indicator of metabolic dysfunction, and the established role of obesity, commonly measured by BMI, as an independent risk factor for cognitive decline (25), the combined indicator of AIP and BMI (AIP-BMI) may provide a stronger theoretical and clinical basis for elucidating the influence of complex metabolic states on cognitive function. In patients with diabetes, elevated AIP levels are significantly associated with an increased risk of cardiovascular events (27). AIP is also positively correlated with the risk of metabolic dysfunction associated with fatty liver disease (MAFLD). The integration of AIP with anthropometric parameters such as waist circumference (WC) and BMI to form the A-W-B model substantially improves the accuracy of MAFLD prediction, offering a novel clinical tool for screening (28). These findings strongly support the notion that combining AIP with individual body composition indices, such as BMI, enables a more comprehensive assessment of metabolic risk. Furthermore, AIP serves as an effective predictor of adverse cardiovascular outcomes. In patients with myocardial infarction with non-obstructive coronary arteries (MINOCA), higher AIP values are closely associated with an increased incidence of major adverse cardiovascular events (MACE), suggesting its potential as a valuable biomarker for non-obstructive coronary artery disease (29). Elevated baseline and cumulative AIP levels are also associated with a higher risk of ischemic stroke, and this relationship varies depending on individual glycometabolic status (14, 20). Among individuals with stages 0–3 of cardiometabolic syndrome (CKM), baseline AIP levels show a significant positive association with stroke risk, indicating its utility for early identification of high-risk populations (19, 30). In addition, both AIP and the triglyceride-glucose (TyG) index independently or jointly affect the risk of all-cause mortality after stroke (21). However, the association between AIP and cognitive function in patients with ischemic stroke has not yet been investigated.

Recent research has extensively examined the relationship between lipid profiles and cognitive function, particularly among individuals with metabolic disorders and older adults. Variations in lipid parameters have been shown to significantly influence cognitive decline. Previous studies indicates that the AIP may serve as a reliable biomarker for assessing the risk of cognitive aging, with its potential predictive value warranting further exploration, especially among younger elderly populations and older women (31, 32). In individuals with a relatively short duration of diabetes (<5 years), lower levels of LDL-C and statin use have been associated with slightly better executive cognitive performance, providing supportive evidence for the role of LDL-C in modulating cognitive risk (33). Among very old Chinese adults, plasma TG levels within the upper-normal range have been positively correlated with preserved cognitive function, whereas lower TG concentrations show no significant association (34, 35). In midlife populations, improvements in specific HDL-C parameters are thought to facilitate cognitive recovery, particularly memory restoration in the early or symptomatic stages of Alzheimer’s disease (AD) (36). Conversely, the LDL/HDL ratio has been closely associated with AD risk, cognitive performance, AD biomarkers, and brain structural integrity, and may influence cognition indirectly through interactions with AD-related biomarkers (37). Interestingly, lower HDL-C levels (quartiles 1–3), compared with higher HDL-C levels (quartile 4), have demonstrated a paradoxical positive association with the maintenance of global cognition and episodic memory. Further analyses revealed that BMI significantly mediates the relationship between HDL-C and episodic memory (mediating effect: 22.2%). This finding suggests that BMI functions not only as an independent risk factor for cognitive decline but also as a key, quantifiable intermediary linking lipid metabolism with cognitive outcomes (38).

However, data on the relationship between AIP and AIP-BMI indices and cognitive function in patients with ischemic stroke remain scarce, and no related studies have yet been published. Our results demonstrate that cognitive function declines markedly with increasing AIP and AIP-BMI levels, underscoring their substantial clinical significance. These findings provide new, integrative evidence supporting the role of the metabolism–cognition axis in post-stroke populations and highlight the synergistic effects of lipid metabolic abnormalities (AIP) and obesity (BMI) in driving PSCI. As an easily calculated composite indicator, AIP-BMI offers strong clinical interpretability and can directly inform risk stratification, enabling the identification of ischemic stroke patients presenting with both dyslipidemia and overweight or obese individuals who may represent a particularly high-risk subgroup for PSCI. This index, therefore, provides a more precise and practical tool for early screening, targeted intervention (e.g., intensive lipid-lowering therapy or weight management), and prognostic evaluation. It also offers a comprehensive framework for understanding the contribution of metabolic risk factors to post-stroke cognitive trajectories and presents new perspectives for future clinical strategies.

Although the precise mechanisms through which the AIP affects cognitive function after ischemic stroke remain not fully elucidated, several plausible pathways may underlie this association. AIP reflects both dyslipidemia and atherosclerotic burden, two well-recognized contributors to stroke and cognitive impairment (15, 39). Elevated AIP levels promote atherosclerotic plaque formation and increase vascular wall instability, leading to diminished cerebral perfusion, microvascular dysfunction, and disruption of the blood–brain barrier. Collectively, these alterations exacerbate neuronal injury and ultimately result in cognitive decline (40–42). These pathological processes may particularly compromise capillary integrity within the hippocampus, a critical region governing memory and learning, thereby further impairing cognitive performance (43). Moreover, the link between atherosclerosis and cognitive dysfunction may be intensified by secondary mechanisms such as chronic hypoxia and systemic inflammation, which amplify neurovascular damage and accelerate cognitive deterioration (44, 45).

Second, numerous studies have demonstrated a significant positive association between the AIP and IR (46, 47). As a hallmark of metabolic dysregulation, IR not only constitutes a central feature of diabetes but is also strongly linked to PSCI. Extensive evidence identifies IR as a key risk factor for cognitive decline and AD (48–50). Mechanistically, IR disrupts cerebral insulin signaling, leading to aberrant lipid metabolism that interferes with neuronal lipid transport and neurotransmitter synthesis, thereby promoting cognitive deterioration (51). Impaired insulin signaling further facilitates *β*-amyloid accumulation and tau protein hyperphosphorylation, amplifying the risk of cognitive dysfunction (51, 52). Among patients with ischemic stroke, the presence of IR may intensify the detrimental effects of glycemic variability and heterogeneous cerebral perfusion, accelerating cognitive decline. Prior studies have also shown that elevated AIP levels markedly increase the risk of incident stroke, particularly in individuals with impaired glucose metabolism (14). Furthermore, research in older adults has revealed that a lower estimated glucose disposal rate (eGDR), indicative of more severe IR, is significantly associated with reduced MMSE scores (53), suggesting that metabolic disturbances may contribute to early cognitive impairment. Collectively, these findings indicate that the interaction between IR and cerebrovascular pathology may synergistically aggravate neural injury, thereby driving progressive cognitive deterioration.

Dyslipidemia can also trigger oxidative stress and lipid peroxidation, during which reactive oxygen species (ROS) attack membrane lipids, leading to cellular injury, neuronal dysfunction, and ultimately cognitive impairment (54). Persistent oxidative stress further disrupts neuronal energy metabolism, diminishes reparative capacity, and accelerates cognitive decline (55, 56). Moreover, dysregulation of HDL-C metabolism may aggravate oxidative injury by weakening antioxidant defenses and promoting neuroinflammation, thereby impairing neuronal growth and repair (57). Experimental evidence indicates that elevated TG levels suppress N-methyl-D-aspartate (NMDA) receptor-mediated synaptic plasticity in the hippocampus, impairing long-term potentiation (LTP) and consequently reducing cognitive capacity (56, 58). LTP, the sustained enhancement of synaptic transmission between neurons, constitutes a fundamental mechanism underlying learning and memory. Elevated TG levels hinder this process, diminishing synaptic signaling efficiency and thereby contributing to cognitive dysfunction. In addition, dyslipidemia is strongly associated with white matter injury. Increased LDL-C and decreased HDL-C levels are frequently accompanied by structural abnormalities in white matter, particularly after ischemic stroke. White matter damage represents a principal pathological substrate of PSCI, characterized by slowed neural conduction and disrupted information transfer pathways, which can result in widespread cognitive deficits (59). The severity of white matter injury correlates positively with lipid abnormalities, suggesting that dyslipidemia exerts a critical influence on neural recovery following stroke. Taken together, these observations, along with the previously discussed associations between lipidomic profiles and cognitive performance, highlight the pivotal roles of dyslipidemia and insulin resistance in modulating cognitive outcomes after ischemic stroke.

As anticipated, modifiable traditional risk factors, such as smoking, hypertension, and LDL-C, remain significantly associated with cognitive function following stroke (25, 26). After adjusting for age and other potential confounders, this study maintained a clear association. Age is a well-established risk factor for cognitive decline post-stroke, with previous studies showing that the risk of PSCI significantly increases in patients aged ≥75 years (60). Based on this, we performed stratified analyses by age, which further confirmed the robustness of our findings. Earlier research indicates that individuals with higher levels of education tend to score better on the MMSE, likely due to education enhancing cognitive reserve, which allows individuals to more effectively mitigate cognitive decline when faced with brain injury (61, 62). These findings align with the results of our study. Hypertension and atrial fibrillation (AF), both common comorbidities in cardiovascular and cerebrovascular diseases, were confirmed to be associated with cognitive decline following stroke in the present study. This conclusion is supported by multiple studies, which indicate that AF not only increases the risk of stroke but is also linked to cognitive decline and an elevated risk of dementia, even in the absence of a visible stroke (63). Similarly, hypertension is closely associated with cognitive decline, with research showing a U-shaped relationship with dementia risk—both high and low blood pressure increase the risk (64). In middle-aged patients with AF, the burden of hypertension control exhibits a linear relationship with dementia risk, suggesting that managing hypertension may help prevent cognitive decline (64). Additionally, the nature of TACI involves extensive ischemic necrosis of brain tissue due to obstruction of the anterior circulation trunk, disrupting critical cognitive networks, including the highly interconnected basal ganglia, limbic, and frontal networks (65), thus contributing to cognitive dysfunction.

### Strengths and limitations

This study is the first to investigate the relationship between AIP, AIP-BMI, and cognitive function after ischemic stroke, offering novel insights and potential therapeutic implications regarding the impact of metabolic indicators on post-stroke cognitive outcomes. However, as a cross-sectional study, it cannot establish causality and may be influenced by unmeasured confounding factors. The focus of this research was on clinical biomarkers related to post-ischemic stroke cognitive function, rather than on molecular or cellular mechanisms. Despite these limitations, the inclusion of multiple confounding factors strengthened the robustness of the findings. The study utilized cross-sectional data, with an average stroke onset of 20 days. It is known that cognitive function fluctuates during the first three months after a stroke. Furthermore, there was no long-term follow-up on cognitive function. Future studies should focus on post-stroke cognitive function at the 3-month time point to further elucidate this relationship.

## Conclusion

This study concludes that elevated AIP and AIP-BMI are independently associated with cognitive decline in patients after ischemic stroke.

## Data Availability

The raw data supporting the conclusions of this article will be made available by the authors, without undue reservation.
