# Supplementary material for: Atherosclerosis index and BMI: new predictors of cognitive function in ischemic survivors
Source: Front Nutr. 2025 Nov 12;12:1703425. doi: 10.3389/fnut.2025.1703425 (PMC12648598; doi:10.3389/fnut.2025.1703425)
Supplement: Supplementary file 1 [file Table_1.DOCX]

**Names of participating research units**

1.First Teaching Hospital of Tianjin University of Traditional Chinese Medicine，National Clinicat Research Center for Chinese Medicine Acupuncture and Moxibustion; 2. Tianjin Academy of Traditional Chinese Medicine Affiliated Hospital; 3. The First Affiliated Hospital of Guangzhou University of Chinese Medicine; 4. The Second Affiliated Hospital of TIANJIN University of Traditional Chinese Medicine; 5. Jiangsu Second Chinese Medicine Hospital(The Second Affiliated Hospital of Nanjing University of Chinese Medicine); 6. Xi'an Hospital of Traditional Chinese Medicine(Xi'an Affiliated Hospital of Shaanxi University of Chinese Medicine); 7. Jingsa Province Hospital of Chinese Medieine(Affiliated Hospital ofNanjing University of Chinese Medieine); 8. Xiamen Hospital of Traditional Chinese Medieine; 9. Tianjin Huanhu Hospital(Tianjin Neurological Central Hospital); 10. Shandong Provincial Hospital Affiliated to Shandong First Medical University; 11. The Third People's Hospital of Jinan; 12. Lianyungang Hospital of Traditional Chinese Medicine; 13. Affiliated Hospital of Shandong University of Traditional Chinese Medicine; 14. the Second Affiliated Hospital of Baotou Medical College; 15. Tianjin Wuqing District Hospital of Traditional Chinese Medicine; 16. Chifeng Hospital of Mongolian Medicine and Traditional Chinese Medicine; 17. Joint Logistic Support Force of the People' s Liberation Army Tianjin Rehabilitation Center; 18. Ningcheng County Hospital of traditional Chinese medicine and Mongolian medicine; 19. Ningqiang County Hospital of Traditional Chinese Medicine; 20. Wuhan Hospital of Traditional Chinese Medicine; 21. the Third Affiliated Hospital of Changchun University of Traditional Chinese Medicine; 22. The First Hospital of Hunan University of Chinese Medicine; 23. Hanzhong Traditional Chinese Medicine Hospital; 24. Haikou Hospital of Traditional Chinese Medicine; 25. Chifeng Municipal Hospital; 26. the 983rd Hospital of Joint Logistic Support Force of PLA; 27. Linyi Hospital of Traditional Chinese Medicine.

**Disease diagnosis criteria**

Hypertension is defined as a clinic measurement of systolic blood pressure (SBP) > 140 mmHg and/or diastolic blood pressure (DBP) > 90 mmHg, or the use of antihypertensive medications[1]. Atrial fibrillation (AF) is defined either by a baseline electrocardiogram (ECG) confirming AF or by a documented history of the condition. The diagnostic criteria for diabetes are based on the standards established by the American Diabetes Association [11]. Specifically, diabetes is diagnosed if, during any follow-up visit, the fasting blood glucose level is ≥7.0 mmol/L, the glycated hemoglobin (HbA1c) level is ≥6.5% (48 mmol/mol), or the patient is receiving antidiabetic medications or insulin. The diagnosis of coronary artery disease (CAD) adheres to international consensus guidelines [3].

[1]Williams B, Mancia G, Spiering W, Agabiti Rosei E, Azizi M, Burnier M, Clement DL, Coca A, de Simone G, Dominiczak A, Kahan T, Mahfoud F, Redon J, Ruilope L, Zanchetti A, Kerins M, Kjeldsen SE, Kreutz R, Laurent S, Lip GYH, McManus R, Narkiewicz K, Ruschitzka F, Schmieder RE, Shlyakhto E, Tsioufis C, Aboyans V, Desormais I; ESC Scientific Document Group. 2018 ESC/EHS Guidelines for the management of arterial hypertension. Eur Heart J 2018;39:3021–3104.

[2]American Diabetes Association. Diagnosis and classification of diabetes mellitus. Diabetes Care 2014;37(Suppl. 1):S81–S90

[3]Fihn SD, Blankenship JC, Alexander KP, Bittl JA, Byrne JG, Fletcher BJ, Fonarow GC, Lange RA, Levine GN, Maddox TM, Naidu SS, Ohman EM, Smith PK. 2014 ACC/AHA/AATS/PCNA/SCAI/STS focused update of the guideline for the diagnosis and management of patients with stable ischemic heart disease: a report of the American College of Cardiology/American Heart Association Task Force on Practice Guidelines, and the American Association for Thoracic Surgery, Preventive Cardiovascular Nurses Association, Society for Cardiovascular Angiography and Interventions, and Society of Thoracic Surgeons. Circulation. 2014 Nov 4;130(19):1749-67. doi: 10.1161/CIR.0000000000000095.

**Table S1**. Association of AIP and AIP-BMI with MMSE (sensitivity analysis)

|  | model1 |  | model2 |  | model3 |  |
| --- | --- | --- | --- | --- | --- | --- |
|  | β(95%CI) | p | β(95%CI) | p | β(95%CI) | p |
| AIP | -0.70(-1.12 to -0.29) | ＜0.001 | -1.16(-1.57 to -0.76) | ＜0.001 | -1.05(-1.45 to -0.65) | <0.001 |
| T1 | ref |  | ref |  | ref |  |
| T2 | -0.70(-1.36 to -0.04) | 0.04 | -1.24(-1.88 to -0.60) | ＜0.001 | -1.14(-1.76 to -0.51) | <0.001 |
| T3 | -0.95(-1.61 to -0.30) | 0.005 | -1.72(-2.37 to -1.07) | ＜0.001 | -1.56(-2.20 to -0.92) | <0.001 |
| P for Trend |  | 0.005 |  | ＜0.001 |  | <0.001 |
| AIP-BMI | -0.02(-0.04 to -0.01) | 0.005 | -0.04(-0.06 to -0.03) | ＜0.001 | -0.04(-0.05 to -0.02) | <0.001 |
| T1 | ref |  | ref |  | ref |  |
| T2 | -0.86(-1.52 to -0.20) | 0.01 | -1.37(-2.01 to -0.73) | <0.001 | -1.26(-1.89 to -0.63) | <0.001 |
| T3 | -0.82(-1.48 to -0.16) | 0.01 | -1.65(-2.30 to -1.01) | <0.001 | -1.48(-2.12 to -0.84) | <0.001 |
| P for Trend |  | 0.015 |  | <0.001 |  | <0.001 |

Model 1: unadjusted; Model 2: adjusted for age, education, smoking status, LDL-C, and duration of illness; Model 3: further adjusted for family history of HTN, the presence of HTN, AF, and OCSP classification (TACI and LACI).


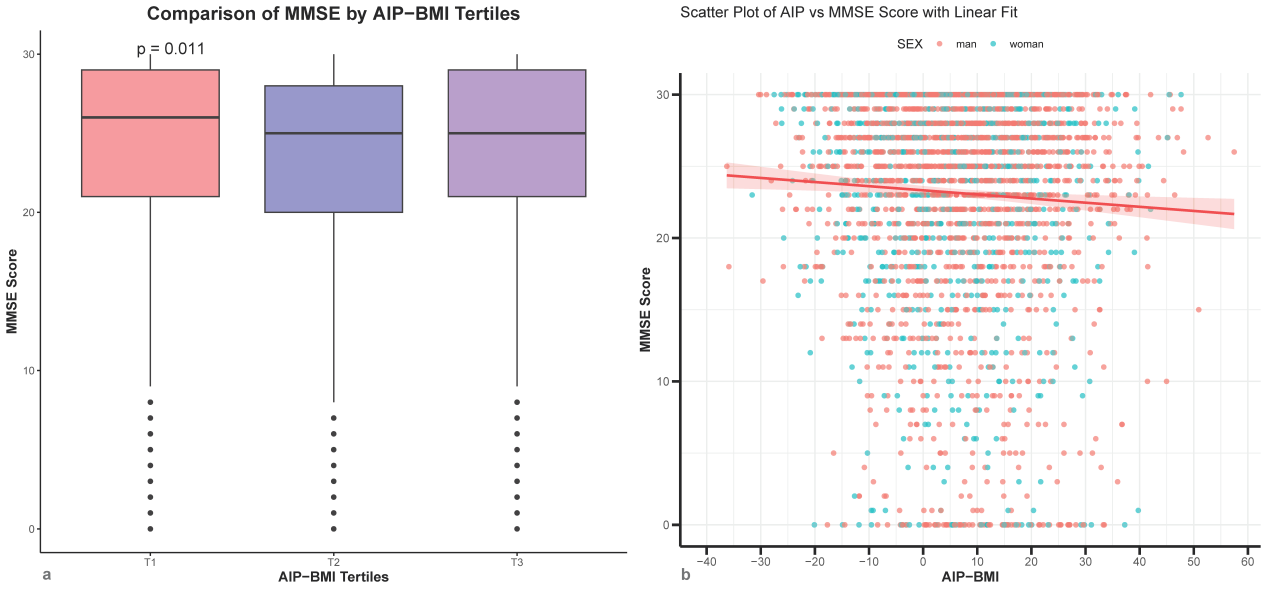


Figure S1a Comparison of MMSE Scores by Tertile Groups of the AIP-BMI. Figure S1b Scatter Plot Showing the Relationship Between MMSE Scores and AIP-BMI in Patients with IS.
